# RNA and proteins joined up at the Origins of Life: Persistence is the point

**DOI:** 10.64898/2026.07.09.737588

**Published:** 2026-07-11

**Authors:** Mohamed Swailem, Ken A. Dill

**Affiliations:** 1 Laufer Center for Physical and Quantitative Biology, Stony Brook University, Stony Brook, NY 11794; 2 Department of Physics and Astronomy, Stony Brook University, Stony Brook, NY 11794; 3 Department of Chemistry, Stony Brook University, Stony Brook, NY 11794

## Abstract

What drove nucleic acids (NA) to associate with proteins (PR) at the Origins of Life? We reason from polymer physics and the Central Dogma (CD) that the fitness value of cooperating through a division of labor – NA for replication fidelity and PR for functional fitness – is much higher than for either polymer alone. Our model shows a Pareto Front, where NA and PR can bootstrap each other to achieve autocatalytic cooperativity towards biology.

To understand how the first cells arose from prebiotic chemistry requires making sense of what drove the association between protein molecules and RNA or DNA molecules (1, 2). Today’s living systems depend on both types of sequence-defined polymers: nucleic acids (NA) (RDNA, our shorthand for RNA and DNA) and protein molecules (PR). We call this a 2-polymer world (2PW). Could a simpler, or earlier, form of life have been a one-polymer world (1PW)? For example the *RNA World Hypothesis* says that because RNA molecules can both carry information and catalyze some reactions, RNA alone might have initiated the first life (3–6). Alternatively, a Proteins World could have produced diverse functionalities prior to heritability (7–14).

The puzzle is framed by the Eigen Paradox (15, 16), which we paraphrase as: *Good RNA is needed to make proteins but good proteins are needed to make RNA*. So naively, it would seem that originating a 2PW would be “twice as improbable” as a 1PW. However, that logic neglects *fitness*, where some PR–NA association has greater advantage than either individual polymer type alone. Here, we model the emergence of the two-polymer association. Reasoning from the Central Dogma of Molecular Biology and polymer physics, the model shows that the fitness advantage of separation of labor – proteins impart functional fitness while RDNA imparts heritability – is so high that the event of their joining together could define the Origins of Life. To set the stage for our modeling, we first consider how the nature of evolution constrains possible relationships between the two polymer types.

## Biological evolution is the context that defines fitness

**Figure 1:**
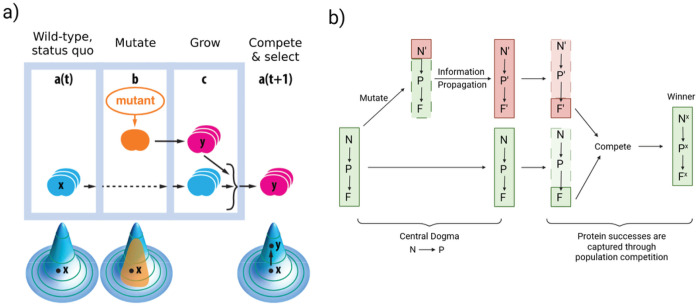
a) Biological evolution as stochastic optimization: Initially, wild-type cells x grow up a population. A mutant y grows up a population. The populations x and y compete. The winner (greater population) then fetches more resources. Fitness landscapes at the bottom show how the mutant is an experiment, where the winner decides the next step direction. b) Evolution annotated in terms of the Central Dogma: (Left, green) Wildtype starting point, NA encodes PR, which gives fitness F. (Top, red), Mutation in the blueprint NA, becoming NA’. (Red, green) both NA sequences grow up into populations. (Next) The populations compete for fitness. The winner, F^x^ now carries with it the winning sequence NA^x^ and protein PR^x^.

Today’s biological evolution is a process of *stochastic optimization* performed by molecules. Fig. 1 shows evolution’s process: (1) Given a wild-type population x, mutate a cell, (2) grow up the mutant into a population y, (3) compete x vs. y for their relative abilities to grow and reproduce, (4) the winner gets more resources going forward into the next cycle. The relative ability to win additional resources is the *fitness*. Below, we first describe how the different physics of RDNA and proteins serve well the two different functions – namely to *explore* and *exploit* – needed for such optimization. Then, we model how their joint W.

## The Central Dogma gives the relationship, *NA* → *PR*

**Figure 2:**
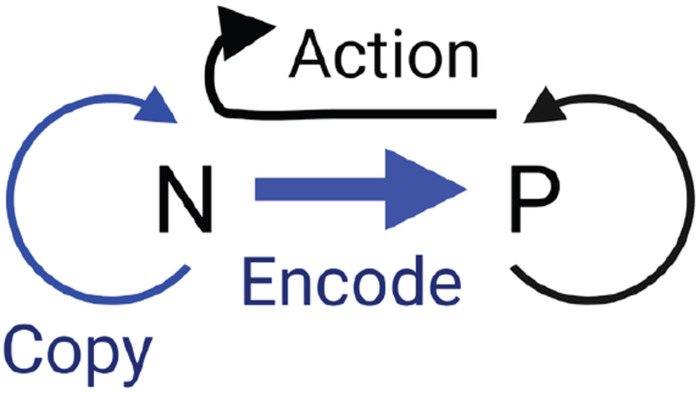
Central Dogma defines a directionality. Information flows in one direction, namely from NA to PR. (Blue arrows) indicate information flow: NA is copied onto NA, and NA encodes PR. (Black arrows) indicate procedural actions that assist in the making process, without changing the sequence.

The *Central Dogma of Molecular Biology* (CD) expresses a master-slave relationship, *NA* → *PR*, of information flow between the two polymer types; see Fig. 2. First elucidated by Francis Crick around 1970 (17), the Central Dogma is not simply that DNA makes RNA makes protein. Rather, Crick distinguished the *informational flow* from the *mechanistic actions* (18). The CD states that once heritable information flows into proteins, it cannot flow back out of proteins.

In Fig. 2, the blue arrows show the two types of *information passage* among molecules: (i) RDNA makes copies of itself and (ii) RDNA act as blueprints that encode proteins. In contrast, the black arrows show *process actions*: both RDNA and proteins act to assist as machinery in making more RDNA and more proteins but do so in a way that is agnostic to – and non-interfering with – the information encoded in the particular sequence.

Fig. 1b gives more mechanistic detail. It shows *NA* as the master and *PR* as the slave, resulting in fitness *F* of the wildtype. Information flows from *NA* → *PR*, and not the reverse. Mutations occur directly in the RDNA, which then gets manifested in proteins. If errors occur in PR, they are not then conveyed to NA.

To illuminate the CD mechanism, here is a metaphor. In an assembly line, the blueprint (nucleic acids) direct the making of product (proteins), resulting in some cost-benefit fitness F for the assembly line of the “factory” (the cell). Changes to the assembly line are performed in a particular way: First a random alteration in the blueprint from NA to NA′ is proposed; that directs an altered product from PR to PR′ ; and that, in turn, gives an altered fitness from F to F ′ . Each assembly line has its own blueprint attached to it at each step of the process. Then there is selection of whichever of the two assembly lines, call it *x*, has the better fitness, F*^x^* . That cycle of evolution/innovation repeats. Each step of each cycle carries “full documentation”, i.e. its own blueprint NA*^x^* and its own PR*^x^*. Once the better assembly line is chosen, its blueprint is already carried along; see Fig. 1b. An alternative procedure would be to edit the blueprint *after* the winner is determined; that would allow for Lamarckian evolution see SI, but would violate the Central Dogma and is not consistent with Darwinian evolution.

The Central Dogma is a statement about today’s biology, not necessarily about how biology arose, or why, or if there are alternatives. But below, we argue that the CD relationship likely reflects important underlying physical constraints on how stochastic optimization can be implemented using molecules.

## Evolution’s stochastic optimization: a division of labor

The Darwinian evolution process may have emerged from something different and simpler, but ultimately the better stochastic optimization process will win out over worse ones. Examples of stochastic optimization computer algorithms are Monte Carlo, simulated annealing, and genetic algorithms. The Metropolis Monte Carlo (MMC) algorithm: make a random change to a state; compare it to the original; select the better of the two. There are two types of stochastic optimization processes: (i) when a fitness function and its gradient are available *a priori* and can be exploited by the optimization procedure or (ii) when it is not known, and must be discovered by trial and error. Evolution is of the latter type; fitnesses become known only through competitions and selection.

One of the deep principles of stochastic optimization is its separation into distinct actions, information-gathering and information-using components, called *explore* vs. *exploit*, or *fluctuations* vs. *drift or bias*. Consider the MMC method. There are three components in taking a step: **(i) Propose:** A uniformly random “dice roll” chooses a possible step even-handedly drawn from a full space of alternative options. **(ii) Compete:** That potential step is then compared to the current state, the status quo value of the property of interest. **(iii) Select:** Accept the step or not based on a criterion of fitness or energy or cost-benefit function of some kind. These three components entail a division of labor that is essential to its success; namely of an *ecumenical* action (universal, even-handed, unbiased) versus a *parochial* action (specific, deciding, choosing, imparting bias). **(1)** **The** ***propose step*** **must be ecumenical/unbiased**, showing no preference of one proposed option over any other. Every sequence in the whole space must be a possible option able to be considered without bias relative to every other sequence, otherwise the process would not be maximally innovative and exploratory. **(2)** ***The select step*** **must be parochial/biased**, meaning that it is evaluative, testing a specific fitness task, different for different sequences. The select step must strongly depend on the fitness landscape starting from the given point of the wild-type organism, while the propose step must be as completely independent of the landscape shape as is possible. We argue below that today’s biology is able to separate ecumenical from parochial actions by parsing different actions into the two different polymer types, NA and PR, because of their very different polymer physics.

## Stochastic optimization implemented in polymers

**Figure 3:**
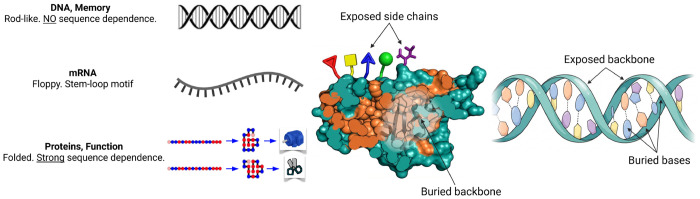
The polymer properties are quite different in DNA, RNA and proteins. DNA molecules are good at unbiased information storage, because the stiff double helical structure is agnostic to sequence and because their bases are buried inside, away from influence by external factors. RNAs, as mRNAs are mostly floppy, not folded, well suited for carrying short stretches of sequence. Proteins are good at function because: they fold; folds are functional; they have 20 different chemical reaction moieties in the sidechains; and the sidechain gardens are exposed externally.

Biology’s sequence polymers are suited for their different roles in this process; see Fig. 3.

- **DNA is ECUMENICAL, well-suited for read/ write/ memory/ bookkeeping.** DNA is stiff. As a form of matter, DNA can carry any arbitrary sequence with minimal conformational variance across sequences, because of its double-helix structure. Conformational energy differences are small for different sequences. And since DNAs bases are buried in the interiors of the double helix, the base sequences are relatively isolated from external environment effects. So, DNA is an ideal blueprint material, for allowing maximal unbiased coverage of sequence space. In contrast, proteins are unreadable; they would be poor as blueprints. Because most proteins are folded, trying to unfold and read them would be like trying to read a book that has its pages glued shut. And, consider the extra challenge that since different proteins have different folded states, it would be challenging to create a single reading machine that could handle all protein sequences without favor.
- **Proteins are PAROCHIAL, well-suited for encodable functionalities/ actions/ growth/ differential populations.** As a form of matter, amino acid sequences each encode different native structures, to accomplish different particular protein functions. Even random libraries of protein sequences exhibit a large space of “biology-like” folded structures (11–13, 19). Different structures participate in different reactions within the cell, making it impossible to replicate protein sequences directly with a singular process. This is the basis for competition and selection on phenotype.
- **RNA is NEITHER.** Single-stranded RNAs are not ecumenical; their conformations depend on their sequences. They are also not parochial, because as a form of matter, most RNA molecules are floppy: they have 7 backbone degrees of freedom between monomers, vs 3 in proteins. RNAs typically have bumpy folding landscapes with many stable and metastable structures (20, 21). Less than 0.5% of the molecular structures in the Protein Data Bank are of RNA molecules. Many RNAs are mRNAs, which are small floppy information carriers from the DNA repository to the ribosomal protein makers. RNA molecules mostly don’t fold or perform protein-like functions, for several reasons; see Fig. 3: (i) RNAs have mainly only 2 types of base pairs, while proteins have 20 different chemistries in their sidechains. (ii) RNA interactions are *internal*, *in base pairing*, not *external, interacting with the outside world* like proteins (Fig. 3). (iii) Because they tend to have multiple metastable states, a sequence doesn’t map to a single structure (22–24). (iv) RNA folding is dominated by secondary interactions favoring linear low-branching stem-loop motifs (25). The most common RNA functional molecules in most cells are ribosomal RNAs or tRNAs, both of which depend on protein partners.
• **POLYSACCHARIDES are not linearly encodable.** Sugar chains can carry information in their sequences of monomer types. But, they also have additional degrees of freedom – branching, linkage position and anomeric configuration (*α* or *β*). So, unlike RDNAs, polysaccharides have no template-based way of forming a linear information pipeline that can store → recover → copy. And unlike proteins, polysaccharides mostly don’t have unique folds and functions because of their hydrophilic character. So, sugar chains are neither ideal for read/write or folding/function actions.

### Origins was a 2-polymer world.

So, summarizing and simplifying: Linear sequence-defined polymers NA and PR are well-suited to different roles in stochastic optimization. DNA is good at read/write/copy/memory, but not folding and function. Proteins are good at folding and function, but poor at read/write/information storage. These virtues coincide with the need for a sequence-independent ecumenical *mutate/propose* step and a parochial *select* step.

### Origins was a transition from *macro* to *micro* catalysts.

Life may have begun on a geographically localized macroscale catalyst that performed a particular reaction under given conditions – a volcanic vent, black smokers, a warm little pond, or a mineral or clay surface, for example (26–29). Euphemistically we call this macro fountainhead “a Founding Rock in Nebraska”. However, the transition to biology arguably required instead catalysis at the microscale – today mainly by protein molecules – that is molecualar, mobile, editable for different catalytic actions and tailorable for different conditions. Below, we reason that the transition to such microscopic fountainheads required both RDNA and proteins, for both heritability and functional variability, perhaps in biocondensates or vesicles or droplets.

### Origins was a transition of timescales, from individuals to lineages.

Chemistry to biology was a transition of timescales. In chemistry, *individual molecules* decay, degrade, dilute or relax to equilibrium on molecular timescales. In contrast, evolution’s timescale is not lifetimes of individuals; it is the lifetimes of *lineages* of individuals and their progeny. Lineages are long-lived because of heritable propagation of short-lived individuals who come and go into or out of the lineage. Individuals are interchangeable. Lineages are about heritability. Oil droplets can divide, but they have no identifiable ancestry, hence no lineages. The transition from chemistry to biology was a transition from short molecule persistence times to long lineage persistence times.

## The transition from chemistry to biology requires *cooperativity*.

Non-equilibrium systems, like living organisms, are often treated by birth-death dynamics, where a population *P* can be described by an equation dP/dt=g(P)P−DP wherein g(P) is the growth rate and D is the death rate. In a prebiotic version of this, there must be some process of molecules making more molecules; and decaying, breaking down, or diffusing away at a total rate D. The simplest starting point is a linear model, having a constant growth rate $g(P)=g_{1}$ (a linear growth function), giving the following dynamics:

(1) $$\frac{dP}{dt}=\left(g_{1}-D\right)P.$$

Consider three regimes of this dynamics. **(1) In prebiotic chemistry,**D wins and $\left(g_{1}-\right.$ D)<0. **(2) In biology,** $g_{1}$ wins, provided a sufficient birth rate, and $\left(g_{1}-D\right)>0$ **(3) For the transition from chemistry to biology,** this linear model is evidently not sufficient to lead to a change the sign of $\left(g_{1}-D\right)$ along some reaction coordinate. It has been shown through *invasion analysis* (8) that the most conservative, lowest-order expansion that can explain a transition from death-dominated to growth-dominated is the following model,

(2) $$\frac{dP}{dt}=\left(g_{1}+g_{2}P\right)P-DP=\left(g_{1}-D\right)P+g_{2}P^{2}.$$

**Figure 4:**
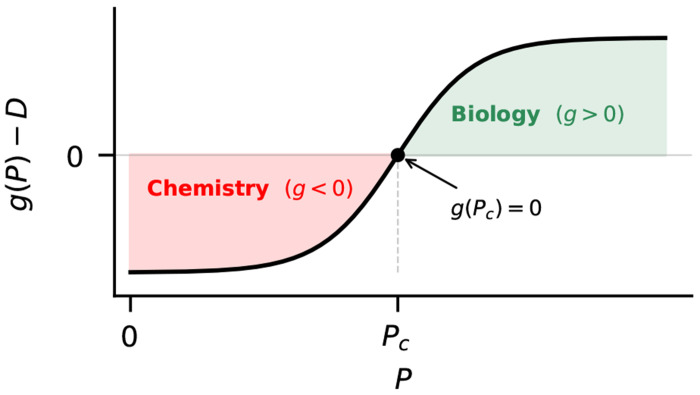
Cooperativity is required for the chemistry to biology transition: How the growth function (g−D)(P) depends on population P, which acts here as a reaction coordinate from chemistry to biology. (Left, prebiotic chemistry,) g−D<0, degradation dominates over growth. (Right, biology,) g−D>0, growth dominates over death. The transition requires cooperativity whereby a mechanism changes how the birth-death dynamics depends on population P.

We refer to the $g_{2}$ term as *cooperativity, contingency, conditionality* or *snowballing*. Eq. (2) is not a mechanistic description; it is just a statement of what population dynamics would be required to transition from one regime (prebiotic, where death dominates) to another (postbiotic, where births dominate); see Fig. 4. In order to be a viable explanation of the transition to biology, a mechanism must do more than simple autocatalysis, more than just each dynamical agent making copies of itself. Along a reaction coordinate from chemistry to biology, there must have been some super-catalysis, some growing advantage of growth vs death terms. Below, we model how RDNA–protein association could originate a Central Dogma relationship by combining heritability or RDNA with function and fitness of proteins.

## Proteins have persistence through folding & catalysis

**Figure 5:**
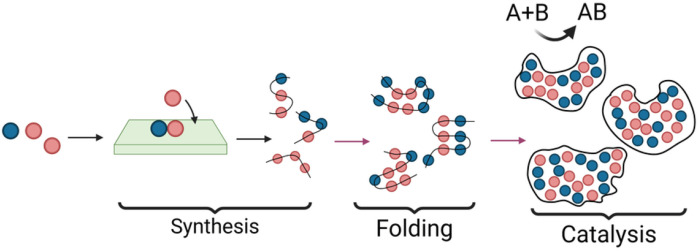
Foldcat mechanism: 1) Random HP chains are synthesized on a ”founding rock”. 2) Short HP chains fold into stable structures due to hydrophobic collapse. 3) A subset of those folded structures serve to catalyze reactions by virtue of localized collections of sidechains.

First consider the protein component. What dynamical mechanism could explain the cooperativity/snowballing (i.e. the $g_{2}$ term) for producing elongating protein chains from amino acids. We adopt here the *Foldcat mechanism* (7, 9, 10). We take as given that amino acids could have been on the early earth (30–32), and that they could have undergone covalent linkages into short random peptides by prebiotic catalysts (33–35). While typical polymers have exponentially diminishing populations with chain length, the Foldcat Hypothesis shows in computer modeling that heteropolymers, such as proteins, composed of hydrophobic (H) and polar (P) monomers, could elongate by two forms of cooperativity. Even random short HP chains will collapse into compact structures in water, reducing the degradation rates of their cores. And at a primitive level, such peptides could catalyze reactions such as chain elongation; see (Fig. 5), contributing to the *g_2_* cooperativity term needed for advancing towards longer-chain biology (8). Importantly, it defines an early form of *fitness*, applicable to molecules in a prebiotic world; namely their relative *persistence* in the environment. If an ecumenical mechanism produces different monomer sequences, those molecules will have differential abilities to fold and catalyze, and not degrade, and thus persist longer and be more available to develop further.

But a proteins-only world is not sufficient to escape to biology. The Foldcat mechanism alone never leads to lineages of multi-molecule complexes. Also, the longer the protein chains become, the more difficult it is to further extend that chain because their folding buries their terminal ends, preventing further extension. At this point, it is important to distinguish what happens to a given HP sequence i, and its population $P_{i}$, versus the population *P* of all sequences in the sequence space, such as in Eq. (2). Any single sequence will die out at death rate D by degradation and dilution (see SI), with dynamics

(3) $$dP_{i}/dt=-D_{P}P_{i}$$

The mean lifetime of any specific sequence is proportional to 1/*D_P_* , while the total population is governed by equation 2 and is able to exhibit cooperativity toward biology. Now consider how partnering of HP foldamer chains with RDNAs can give a huge advantage of fitness as persistence.

## RDNA gives fidelities, but lacks selection.

A mechanism of RDNA would entail replication. As in the case with proteins above, we label sequences by the index i. Sequences have a birth and death rate, and its birth is dependent on having a template. However, because RDNA is ecumenical, no one sequence has any advantage over any other. For an RDNA-only world, each RDNA sequence has birth-death dynamics (see SI for details):

(4) $$\frac{dR_{i}}{dt}=\mu \kappa R_{i}-D_{R}R_{i},$$

where κ is the replication speed, the doubling time of an RDNA population, having units of 1 /time. And μ is the probability that a sequence makes an exact copy of itself (the replication fidelity), a dimensionless quantity representing the accuracy of correctly attaching a correct Watson-Crick pair versus an incorrect pair. In Eq. (4), what matters is only the product μ×κ, but below we need these two quantities separately. Finally, we note that in accord with our assumption above that the replication machinery itself must be agnostic to what sequence it is replicating, κ does not have an index *i*.

In this model, RDNA is capable of self-copying, but has no mechanism for fitness/ selection, because copying is assumed to be ecumenical, the same for all sequences. In addition, an RNA-world that contains only individual molecules lives only on a molecular timescale, since molecules degrade with prebiotic rates (36, 37).

## A model for the emergence of PR-NA coupling

**Figure 6:**
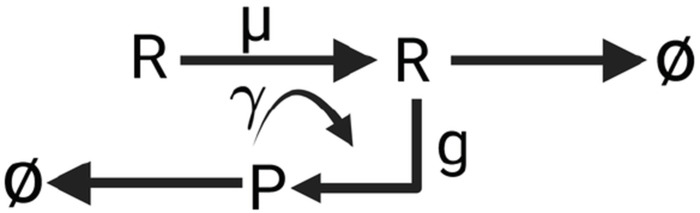
Protein-RDNA CD model: µ is a heritability order parameter for how well RDNA can copy itself. γ is a functionality/ catalyst order parameter for how well proteins assist heritable copying. g is the rate at which RDNA molecules produce protein.

Based on the reasoning above, we assume a separation of actions whereby peptides fold and function as in the Foldcat model and proteins aid the replication of RDNA; see Fig. 6. This requires that RDNA can support protein synthesis; some such actions have been observed experimentally (38–41). Our mechanism has cooperativity: The larger the protein population, the better the RDNA replication. Our model does not explicitly address the microscopic basis of this cooperativity, but various potential sources could be considered: activation of substrate; (42); positively charged proteins can assist the polymerization of negatively charged RDNA (24); of the electrostatic forces that tend to localize and stabilize

RDNA-Protein biocondensates (43). We don’t seek a more granular mechanism here, in order to avoid speculating and additional parameters that would obscure the main points here.

Assuming the two polymers have a CD relationship gives the following dynamics:

(5) $$\frac{dP_{i}}{dt}\;=gR_{i}-D_{P}P_{i},$$

(6) $$\frac{dR_{i}}{dt}\;=\left[\left(\kappa +\gamma_{i}P_{i}\right)\mu -D_{R}\right]R_{i}.$$

where $P_{i}$ and $R_{i}$ are the protein and RDNA populations respectively ad the index i refers to individual sequences of RDNA and proteins. The coupling parameters g and $D_{P}$ represent the rate of protein synthesis and degradation, and γ represents the protein fitness for the job of replicating any RDNA on a template.

Here are some properties of the model. When g=0, when proteins are not encoded by the RDNA, the protein population decays to zero. That situation reduces to an RDNA-only world. It does not exhibit cooperativity, all the RDNA sequences are equivalent, and they have no differential fitness. Instead, γ=0 describes a situation where protein does not aid RDNA replication. This too has no cooperativity or selection. Only when g and γ are both non-zero does the model describe the organization of proteins and RDNA in an interacting assembly. This unit of evolution now has cooperativity since $\gamma_{i}$, which depends on the sequence contributes to RDNA replication. And specific protein sequences are now able to control the growth of the assembly by indirectly controlling the RDNA population $R_{i}$. In contrast, in a protein-only world, each sequence contributes to an overall average fitness $\overset{‾}{k}$, and then the label i is best regarded as the label of a specific assembly of proteins and RDNA, something that is capable of having a lineage, if the order parameters are favorable enough.

A key conclusion is that this mechanism, equations (5) and (6), have the type of cooperativity needed according to Eq. (2) to allow the escape from chemistry to biology. Define $C=\sqrt{P^{2}+R^{2}}$, an effective quantity to whether the assembly grows or decays. In the SI, we performed standard stability analysis and found that a transition from growth to decay appears in our system as a saddle node fixed point. Near the transition the growth/decay dynamics are given by:

(7) $$\frac{dC}{dt}=-\left(D_{R}-\kappa \mu \right)C+\frac{g}{D_{P}}(\gamma \mu )C^{2}.$$

Equation (7) has the cooperativity form of Eq. (2). It corresponds to growth ate $g_{1}=\kappa \mu$, degradation rate $D_{R}$ and cooperativity parameter $g_{2}=(g\gamma \mu )/D_{P}$. The linear growth term is entirely controlled by the RDNA replication parameters, consistent with the Central Dogma principle. Proteins only affect this transition via the parameter γ. Note that the cooperativity parameter is linear in the product γμ if we consider only the lowest-order coupling in a 2PW.

Now, we describe how Eq. (7) predicts a chemistry-to-biology transition. The protein fitness and RDNA fidelity parameters are captured through *γ* (proteins assisting RDNA copying) and *μ* (RDNA copying fidelity), respectively. Therefore, to address the Eigen paradox, we determine the relation between the critical value of these two order parameters required for escape. This is slightly different from the usual Eigen threshold (15, 16), which describes the stability of information in an error-prone replication system. Rather, we focus here on finding the threshold of fitness and fidelity needed for our model protein-RDNA assembly to transition toward biology.

**Figure 7:**
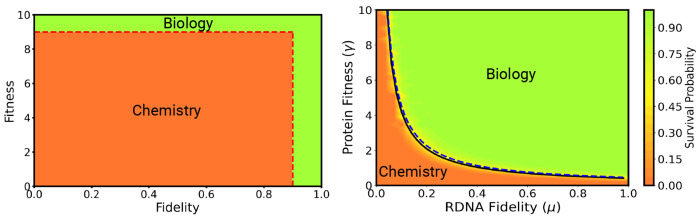
Computed Pareto Front for escaping from chemistry to biology, for the two evolutionary parameters µ and γ. (Left, Eigen box assumption:) both proteins and RDNA must be good independently to escape chemistry. The horizontal red line shows the necessary fitness in a protein-only world with poor fidelity µ = 0.111. The red vertical line is the necessary RDNA fidelity in an RDNA-only world with poor fitness γ = 1.11. (Right, computed phase diagram from the model:) (Red) Both values are small, growth dies out. (Green) The combination of values is large enough that escape to biology is autocatalytic. (Black solid line) The deterministic boundary obtained by numerically solving the system. (Blue dashed line) is our analytical approximation of the Pareto Front boundary.

We modeled the critical catalytic strength *γ_c_* and fidelity *μ_c_* to obtain the transition barrier of survival probability using the Gillespie algorithm and the deterministic boundary is obtained by solving equations (5) and (6) numerically, using the following parameters: $D_{P}=1,D_{R}=0.5,\kappa =0.05,g=0.006$, and $\left(P_{0},R_{0}\right)=(10,10)$. The red region is where proteins and RDNA are not sufficient to escape chemistry; the green region is where the polymers are sufficient to escape. The blue parabolic curve on the left Fig. 7 separating the red from green regions is the tipping line from chemistry to biology. The tipping line curve resembles a Pareto front where low fidelity RDNA can be compensated for by high fitness proteins or vice versa.

The shape of this Pareto front can be obtained analytically by an informal argument. The critical point is determined by the parameters $g_{1}$ and $g_{2}$ in Eq. (2). Since $g_{1}$ doesnť depend on γ or μ, the relation between $\gamma_{c}$ and $\mu_{c}$ can be obtained by essentially fixing the critical $g_{2}\propto \gamma \mu$. Therefore, we obtain the functional form of the Pareto front $\gamma_{c}\propto 1/\mu_{c}$. A more rigorous calculation is given in the SI which also allows the analytical determination of the Pareto front as follows:

(8) $$\gamma_{c}=\frac{A}{\mu_{c}}.$$

where A is a prefactor that depends on the remaining parameters and the initial concentrations $P_{0}$ and $R_{0}$. The exact form of A is given in the SI. The analytical computation of the Pareto front is valid in a prebiotic approximation, wherein protein synthesis is slower than protein degradation (D≫g). However, we find excellent agreement between the analytical solution and the numerical solution as shown in Fig. 7. In general, we expect the inverse relation between $\gamma_{c}$ and $\mu_{c}$ to still hold even if this approximation is not valid, with only a change in the prefactor.

This model is simplified. It is only intended to capture the essence of the two sequence polymers, and assumes the CD relationship shown in Fig. 6. It is not a microscopic mechanism of their association. And while it does assume the dynamics of multi-molecule objects, it leaves out metabolites, cell-like encapsulation and small molecule processes that may have accompanied this 2-polymer association (44–47). We note, however that random RNA- protein associations are not rare events, as is becoming increasingly understood in studies of their biocondensates (43, 48–57). In particular positively charged proteins naturally associate with RNAs, which are negatively charged in solution, across many different compositions, chain lengths and sequences (58).

Comparing the left and right panels in Fig. 7 shows the new insight this model gives into the origins of a 2PW. The right figure shows what we call the Eigen Box. It supposes that good protein is needed to make RDNA, and that good RDNA is needed to make protein, and that the two polymers must achieve those conditions independently. For this figure, the definition of “good” is arbitrarily set to 90% of perfect. Users could choose other values, or sequential mechanisms, one polymer before the other (59). In short, the main point of comparison of the two panels of Fig. 7 is in showing that once RDNA and proteins become coupled – even if each one individually is poor at its job – the 2 polymers can cooperate in a sort of double-bootstrapping: poor-quality RNA can be rescued by good-quality protein or vice versa.

**Figure 8:**
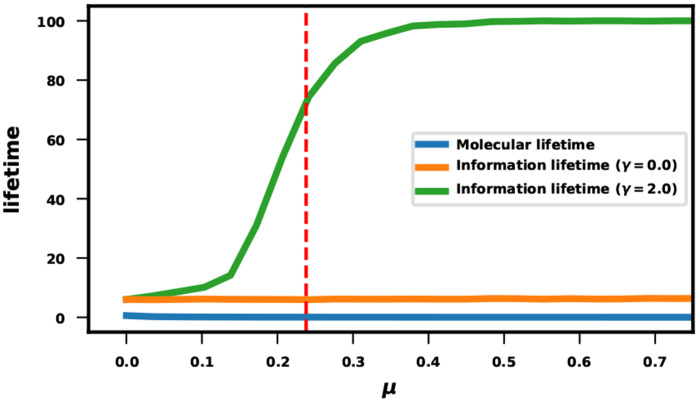
The persistence (fitness) is much greater for Protein-RDNA than for either alone. Plots showing the lifetime of the entire RDNA population (the information lifetime) and the average lifetime of a single RNA molecule in the system as a function of µ for γ = 0 (orange curve), and γ = 2.0 (green curve).

Fig. 8 shows an important conclusion from the model; namely that the joining together of RDNA with protein leads to far greater persistence – and thus far greater fitness – than is achievable by either individual polymer alone. This is because when RDNA and protein combines cooperatively to evolve with some degree of heritability – even with only moderate fidelity – those complexes now have the much longer lifetimes as lineages than the lifetimes that individual molecules alone would have. Fig. 8 shows the different levels of persistence with different model parameters. The curve with γ=0, no protein assistance for RDNA replication, shows little ability to persist for any value of μ, the fidelity parameter. In contrast, the curve with γ=2, representing some degree of coupling, reaches much longer persistence, for sufficiently large µ. In comparison, the molecular lifetime is not affected by the coupling, meaning that this fitness advantage is not due to the increase in the lifetime of any single RDNA molecule, but due to the cooperativity provided by proteins. These plots were generated with an initial population of 6 RDNA molecules and a maximum simulation time of t = 100. Therefore the plateau occurring after the dashed vertical line is an artifact of our finite simulation time.

## Summary

How might prebiotic chemistry have led RDNA and proteins into cooperative functional associations? We give here a model that assumes a separation of labor, proteins acting to assist RDNA replication fidelity, in a Central Dogma arrangement. The model has two evolutionary parameters, allowing us to study the emergence of order from disorder. The model has the type of second-order cooperativity needed to get from chemistry to biology. The two polymers leverage each other to grow to high joint fitness, where lineages now dominate over single molecules. The model predicts a double bootstrapping cooperativity: This 2-polymer world (RDNA, protein) had substantially better ability to persist than either single polymer world alone would have had.

## Supplementary Material

Supplement 1

## Data Availability

The code used to conduct this research can be found in a public github repository at: https://github.com/mswailem/RPCDmodel
